# Hepatic Positron Emission Tomography: Applications in Metabolism, Haemodynamics and Cancer

**DOI:** 10.3390/metabo12040321

**Published:** 2022-04-02

**Authors:** Miikka-Juhani Honka, Eleni Rebelos, Simona Malaspina, Pirjo Nuutila

**Affiliations:** 1 Turku PET Centre, Turku University Hospital, 20520 Turku, Finland; simona.malaspina@tyks.fi (S.M.); pirnuu@utu.fi (P.N.); 2 Turku PET Centre, University of Turku, 20520 Turku, Finland; eleni.rebelos@utu.fi; 3 Institute of Clinical Physiology, National Research Council (CNR), 56124 Pisa, Italy; 4 Department of Endocrinology, Turku University Hospital, 20520 Turku, Finland

**Keywords:** positron emission tomography, fluorodeoxyglucose, liver metabolism

## Abstract

Evaluating in vivo the metabolic rates of the human liver has been a challenge due to its unique perfusion system. Positron emission tomography (PET) represents the current gold standard for assessing non-invasively tissue metabolic rates in vivo. Here, we review the existing literature on the assessment of hepatic metabolism, haemodynamics and cancer with PET. The tracer mainly used in metabolic studies has been [^18^F]2-fluoro-2-deoxy-D-glucose (^18^F-FDG). Its application not only enables the evaluation of hepatic glucose uptake in a variety of metabolic conditions and interventions, but based on the kinetics of ^18^F-FDG, endogenous glucose production can also be assessed. 14(R,S)-[^18^F]fluoro-6-thia-Heptadecanoic acid (^18^F-FTHA), ^11^C-Palmitate and ^11^C-Acetate have also been applied for the assessment of hepatic fatty acid uptake rates (^18^F-FTHA and ^11^C-Palmitate) and blood flow and oxidation (^11^C-Acetate). Oxygen-15 labelled water (^15^O-H_2_O) has been used for the quantification of hepatic perfusion. ^18^F-FDG is also the most common tracer used for hepatic cancer diagnostics, whereas ^11^C-Acetate has also shown some promising applications in imaging liver malignancies. The modelling approaches used to analyse PET data and also the challenges in utilizing PET in the assessment of hepatic metabolism are presented.

## 1. Introduction

### 1.1. Hepatic Physiology

**Figure 1:**
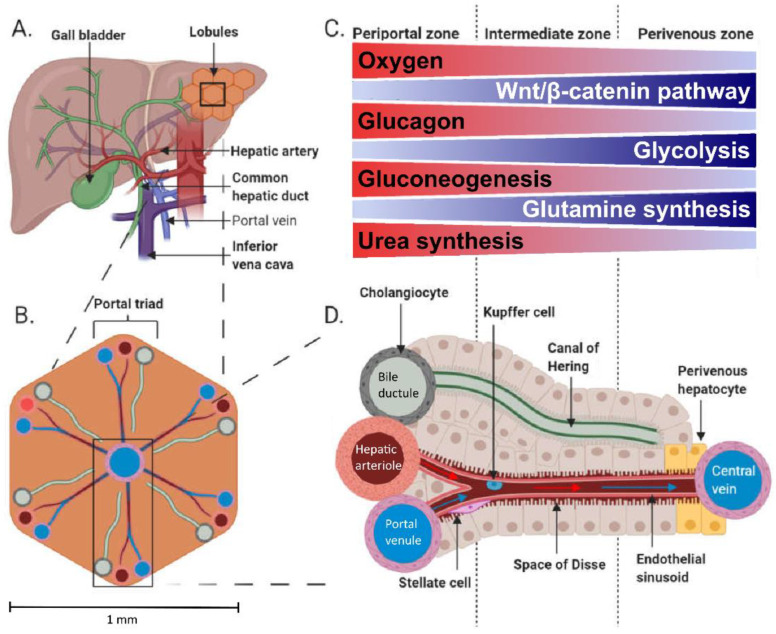
Anatomy (**A**) and physiology of the human liver. The microstructure of the liver consists of small, generally hexagonal lobules (**B**). The portal triads comprising a portal venule, hepatic arteriole and bile ductule are located at the corners of the lobules. The acinus is divided into three zones (**C**), periportal, intermediate and perivenous, with varying environment and metabolic activities according to the location between the portal triad and central vein (**D**). The acinus comprises a sinusoid connecting hepatic arteriole and portal venule with the central vein where proteins and metabolites are exchanged in the plasma-containing space of Dissé. Bile secreted from the hepatocytes drains into a canal of Hering, which empties into a bile ductule. (**A**,**B**) and (**D**) are adapted from [4] and C from [5] under Creative Commons Attribution 4.0 International License (http://creativecommons.org/licenses/by/4.0/, accessed on 17 January 2022).

The liver receives ~29% of cardiac output, and it has a unique perfusion system receiving blood from the portal vein and the hepatic artery. The portal vein provides approximately 75% of the liver’s blood supply, whereas the hepatic artery contributes the remaining 25% [1]. The hepatic artery branches off the celiac trunk, which is the first major branch of the abdominal aorta [2] and provides highly oxygenated blood to the liver. The portal vein provides partially oxygenated blood to the liver [3], and nutrients absorbed from the gut pass first through the portal vein before being released to systemic circulation from the hepatic vein (Figure 1). The portal vein drains through the gastrointestinal tract, spleen and pancreas and is formed through a confluence of the superior and inferior mesenteric veins, splenic veins, pancreatic vein, and left and right gastric veins.

Blood flow through the splanchnic area increases after a meal to distribute the ingested nutrients [6,7] and to compensate for meal-induced increased oxygen use by the splanchnic organs [8]. Portal venules and hepatic arterioles provide blood supply throughout the liver parenchyma and are interconnected into liver sinusoids by anastomoses where arterial and portal blood mix [9]. The microstructure of the liver consists of small, generally hexagonal lobules of ~1 mm diameter in humans (Figure 1B). These lobules are perfused by a portal triad at each corner and a hepatic vein at the centre of the lobule, which are connected by the highly fenestrated sinusoids. The portal triad consists of a portal venule, hepatic arteriole, and bile ductule (Figure 1D). This anatomical architecture is accompanied by functional zonation (Figure 1C). The periportal hepatocytes close to a portal triad are active in gluconeogenesis, amino acid catabolism and urea synthesis, whilst the pericentral hepatocytes with lower oxygen supply have active glycolysis, branched-chain amino acid catabolism and glutamine synthesis [10,11,12]. Some functions such as glycogenesis are more uniformly performed across the lobules [11,13]. Fatty acid oxidation has been generally thought to occur mainly in periportal hepatocytes and fatty acid synthesis in pericentral cells; however, the zonation of these processes seems to be flexible depending on the metabolic state [11,14]. Detailed inspection of fatty acid oxidation in murines has revealed the periportal zone to be the main site for mitochondrial β-oxidation and pericentral zone for peroxisomal β-oxidation [12,15]. Change in hepatocyte oxygen supply is a likely driver of the functional zonation across the liver acinus [16]: The hepatic artery partial oxygen pressure (PaO_2_) is ~95 mmHg, whilst PaO_2_ in the portal vein is ~50–55 mmHg, and the hepatic vein is 30–40 mmHg [3,17], whereas in vivo measured liver tissue PaO_2_ is ~30–55 mmHg [18,19,20]. In addition, the Wnt/catenin, Hedgehog [21], and glucagon signalling [22] have been shown to contribute to the liver’s functional zonation.

The liver represents the major metabolic hub of the human body. In the postprandial state, when there is no need for additional carbohydrates, the liver stores glucose as glycogen or converts it to lipids, whereas in the postabsorptive state, the liver contributes to endogenous glucose production through glycogenolysis and gluconeogenesis and oxidises fatty acids to produce ketone bodies that are used as metabolic fuels in extrahepatic tissues comprising the brain [23]. Liver fatty acid oxidation is high in the fasted state [24,25], whereas fatty acid oxidation declines considerably after a mixed meal/presence of hyperglycaemia [26,27,28]. This, together with knowledge that splanchnic area oxygen consumption increases after a mixed meal [29], means that the oxidations of lactate, amino acids and glucose are preferred in the postprandial state [30]. The liver takes up approximately 25–40% of an oral glucose load [31]. Seventeen to nineteen percent (17–19%) of an oral glucose load has been found to be incorporated to liver glycogen, of which 50–70% is formed via the direct pathway and the rest from cycling through 3-carbon precursors [32,33,34,35]. At the postabsorptive state, the liver produces about 80% of the glucose that appears in the circulation [36]. When the liver’s glycogen stores are full, glycogenolysis is the main contributor to the liver’s glucose production, whereas the relative contribution of gluconeogenesis increases when glycogen stores are used during fasting until glycogen stores are depleted, and about 100% of glucose production comes from gluconeogenesis [37,38,39,40].

### 1.2. Traditional Nodes of Assessing Tissue Metabolism vs. PET

With the arteriovenous (AV) difference technique, the net balance across an organ can be determined. The method is based on measuring blood flow and the concentration difference of the metabolite of interest between an artery delivering the organ and a vena draining from the organ [41]. In the case of glucose-producing organs, the liver and the kidney, the use of glucose isotopes is needed to quantify glucose uptake and glucose production rates. In principle, sampling from an artery and a vein draining the organ of interest is required. As already mentioned, the liver receives blood both from the portal vein and the hepatic artery; the portal vein is virtually inaccessible in human studies; therefore, human studies employing the AV difference technique cannot yield estimates of solely liver metabolism but rather of the splanchnic region [42]. Moreover, the AV determination across organs with very high blood flow (such as the liver and the kidney) represent an analytical challenge. These limitations of the AV difference technique can be overcome with the application of non-invasive imaging techniques such as positron emission tomography (PET) or magnetic resonance imaging (MRI) and spectroscopy (MRS)—which of course have their own limitations. In this article, we will focus on the use of PET in the assessment of liver metabolism, haemodynamics and cancer.

## 2. Studying Liver Metabolism with PET

### 2.1. Principle of PET

PET is a highly sensitive medical imaging technique that is based on measuring the radioactive decay of labelled positron emitting compounds injected into humans or animals. A portion of gamma rays resulting from the annihilation of the emitted positrons proceeds through the body tissues and is measured with the detector ring of a tomograph and computationally constructed to form three-dimensional images of tracer activity. Labelling energy substrates such as glucose or fatty acids with a positron emitting isotope allows the measurement of the localization of positron decay and, thus, the uptake of the tracer compound. 

### 2.2. Study of Hepatic Glucose Uptake Using PET

**Figure 2:**
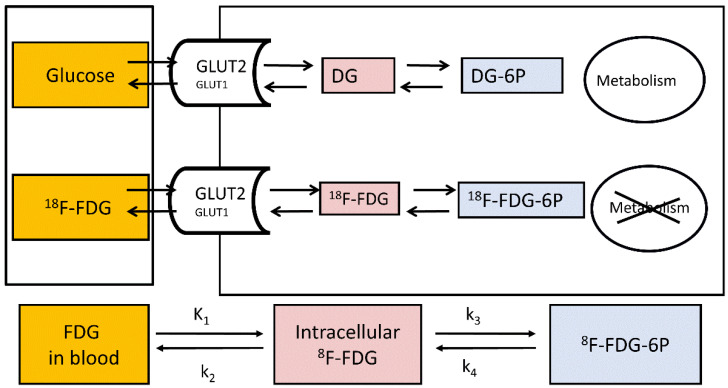
Measurement of hepatic glucose metabolism using 18F-FDG. K_1_: clearance from blood to cell (mL of blood/min/cm^3^ of liver tissue); k_2_: back flux of ^18^F-FDG (min^−1^); k_3_: phosphorylation of ^18^F-FDG to ^18^F-FDG-6-phosphate (min^−1^); and k_4_: dephosphorylation of ^18^F-FDG-6-phosphate to ^18^F-FDG (min^−1^).

A positron-emitting glucose analogue ^18^F-labelled fluorodeoxyglucose (^18^F-FDG) is widely used for the assessment of tissue glucose uptake (GU) with PET in both clinical and research settings, and the euglycemic-hyperinsulinemic clamp allows the measurement of tissue-specific insulin-stimulated GU [43,44]. The structure of ^18^F-FDG is favourable for measuring tissue GU due to its limited metabolism; also, it is a good substrate for the facilitated-glucose transporters responsible for glucose transport to and from the liver [45,46]. ^18^F-FDG transported into the cells is either phosphorylated to ^18^F-fluorodeoxyglucose-6-phosphate (^18^F-FDG6P) or transported back out of the cell. The phosphorylated ^18^F-FDG6P is not transported out of the cells as G6P transporters reside on intracellular membranes and not on cell surfaces [47]. Once phosphorylated, ^18^F-FDG6P does not proceed to glycolysis (Figure 2), and its metabolisms into the glycogen or pentose phosphate pathways are slow in the liver [48,49]. In fact, in a pig study where we validated the use of ^18^F-FDG-PET in the quantification of hepatic GU, livers were biopsied 3h after ^18^F-FDG injection, and no detectable amount of liver ^18^F-glycogen was found [50]. The metabolites 2-^18^F-fluoro-2-deoxy-6-phospho-d-gluconolactone (^18^F-FD6PGL), 2-^18^F-fluoro-2-deoxy-6-phosphogluconate (^18^F-FD6PG1), NDP-2-^18^F-FDG or NDP-2-^18^F-fluoro-2-deoxy-D-mannose may become important in the liver when the scan duration is longer than 60–90 min [49,51,52]. However, ^18^F-FDG6P can be dephosphorylated by glucose-6-phosphatase which works actively in the liver. Nevertheless, (1) dephosphorylation activity is generally low compared to phosphorylation [50,53,54] and (2) the phosphorylation of glucose entering the cells and the dephosphorylation of G6P derived from gluconeogenesis, glycogen breakdown or cycling back from glycolysis are compartmentalized processes [16,50,53], allowing thus the measurement of GU in the liver. Liver ^18^F-FDG6P trapping is enhanced in insulin-sensitive individuals during a hyperinsulinemic clamp where insulin stimulates glucokinase activity while simultaneously suppressing the activity of glucose-6-phosphatase, whereas the suppression of glucose-6-phosphatase activity is blunted in persons with insulin resistance [53].

There are several modelling approaches that can be used to quantify liver glucose metabolism. A dynamic study where tracer concentration is recorded over time in blood to measure tracer input and in target tissue is ideal for quantification. Arterial input function can be precisely measured for PET-studies and arterialised input function corresponds to arterial input reasonably well for ^18^F-FDG studies [55,56]. However, the dual blood input of the liver poses a challenge for human studies as the portal vein is inaccessible and deriving image-based input functions is difficult due to the small diameter of the vein and respiratory motion [57]. Assuming that an input consists of arterial blood only leads to underestimation of compartmental model rate constants [57,58]. Nevertheless, in our previously mentioned validation study in pigs, the underestimation of GU when using only arterial input function was found to be relatively small (5–10%) [50]. This difference is smaller than interindividual variation in hepatic GU and likely of negligible clinical relevance. Despite underestimation when using only arterial input, GU measured with single and dual input were highly correlated (r = 0.998) [50]. Furthermore, several models have been developed to estimate portal vein (dual) input function [59,60]. Only the arterial input function can be used for liver tumour studies because the hepatic artery is the main blood supply for them [61,62,63].

A three-compartment model consisting of blood, intracellular space and phosphorylated state can be used to model GU [56] (Figure 1) using the following equation:

(1) $${\mathrm{MR}}_{\mathrm{glucose}}=\frac{C_{\mathrm{glucose}}}{\mathrm{LC}}\times \frac{K_{1}^{\;*}\times {\;k}_{3}^{\;*}}{k_{2}^{\;*}+{\;k}_{3}^{\;*}}\;$$

where MR_glucose_ is the metabolic rate of glucose (GU rate), K_1_^*^ is unidirectional rate constant of transport from blood to hepatocytes (hepatic systemic clearance), k_2_^*^ is the backflux of tracer from hepatocytes to blood and k_3_^*^ is the rate constant of ^18^F-FDG’s phosphorylation. C_glucose_ is the plasma glucose average from ^18^F-FDG injection until the end of the PET scan. LC is a lumped constant adjusting for differences between the transfer rates of ^18^F-FDG and glucose of the tissue, which is studied. Studies in pigs and humans suggest LC in the liver does not differ from unity [50,64]. However, a different LC might need to be considered if using a compartment model where the dephosphorylation of ^18^F-FDG6P is included (k_4_ > 0) [65,66], depending on the input function used or the presence of a liver disease [67]. In addition, the presence of other metabolites of ^18^F-FDG than ^18^F-FDG6P would cause some underestimation of k_4_ [68]. Importantly, measuring MR_glucose_ does not require blood flow measurements, thus providing an advantage over the arteriovenous difference and microdialysis techniques, which require information about blood flow.

Another way to calculate the GU rate is to use a graphical analysis method: the Patlak plot [69,70]. This analysis assumes that, if there is irreversible tracer uptake, the relationship between tracer activity available in blood and accumulated in tissue becomes linear after the initial mixing of tracer pools reaches an effective steady state [69,70]. The Patlak plot measures the irreversible tracer uptake whereby a net transfer rate is described using term K_i_^*^, which combines K_1_^*^, k_2_^*^ and k_3_^*^.

(2) $$K_{i}^{\;*}=\frac{K_{1}^{\;*}\times {\;k}_{3}^{\;*}}{k_{2}^{\;*}+{\;k}_{3}^{\;*}}\;$$

(3) $${\mathrm{MR}}_{\mathrm{glucose}}=\frac{C_{\mathrm{glucose}}}{\mathrm{LC}}\times {\;K}_{i}^{\;*}\;$$

The presence of dephosphorylation would show as a bend in the Patlak plot over time [64,71]. An important consideration when measuring liver GU is that higher liver fat content reduces the distribution volume of ^18^F-FDG in the tissue (V_0_), thus reducing K_i_. According to some authors [72], this issue can be addressed by dividing K_i_ with the *y*-axis intercept of the Patlak plot because in healthy livers, V_0_ is close to unity, meaning that ^18^F-FDG quickly distributes into both hepatic interstitial space and hepatocytes and K_i_/V_0_ equals k_3_, which describes the conversion of ^18^F-FDG to ^18^F-FDG-6P. Thus, k_3_ calculated this way would describe glucokinase activity in a lean fraction of liver tissue. A simple model for measuring GU rates is the application of fractional uptake rate (FUR), a method that has been validated against the Patlak plot. Moreover, FUR measurements of blood activity are required, but only one timepoint of tissue activity (static image) is enough to measure tissue activity. With this model, the average tissue activity is divided by the integral of the plasma input activity from the tracer’s injection to the middle time of the PET frame used in FUR calculation, and the effective distribution volume with FUR is assumed to be 0 [73]. FUR yields an estimate of the tracer net transfer rate, which can be translated to a GU rate by multiplying it with the plasma glucose average and dividing by a lumped constant (similarly to the K_i_ obtained from the Patlak plot). Using FUR leads to some overestimation of the true net transfer rate. The bias is over 20% within the first 20 min from ^18^F-FDG injection and becomes less than 5% after 60 min of scan time [74]. Therefore, FUR is an accurate alternative for the Patlak plot when only static images are available, and the scanning time is long.

The measurement ofa standardised uptake value (SUV) is a semiquantitative method for assessing tissue GU. An SUV is calculated by multiplying tissue activity with body weight and dividing the result by the injected dose. SUV is affected by several factors, which can lead to large errors and hamper the use of SUV for the measurement of glucose metabolism: (1) body composition and habitus; (2) length of uptake period; (3) plasma glucose; (4) recovery coefficient and partial volume effects (these affect Ki and FUR, too) [75]; and (5) variable urinary excretion [76] or a sink effect caused by variable tissue uptake (e.g., high tumour uptake) on blood tracer concentrations, which affects the total tracer clearance [77]. From these, body fat content and the length of the uptake period are major sources of variation [75]. Although these matters can be often addressed by standardizing scanning times, correcting for anthropometric features and plasma glucose, the accuracy of SUV is inferior to quantitative three-compartment modelling, the Patlak plot and FUR [78]. Using an SUV ratio (SUVR), i.e., adjusting tissue SUV by blood improves the accuracy of an SUV measurement [79] because it accounts for possible variations in tracer supply.

### 2.3. Evaluation of Endogenous Glucose Production during ^18^F-FDG-PET

The use of ^18^F-FDG for the measurement of PET GU allows simultaneous measurement of endogenous glucose production (EGP) at both fasting states and during a euglycemic-hyperinsulinemic clamp [44,80,81]. This measurement is based on determining glucose disappearance rate from plasma by calculating ^18^F-FDG clearance, which is corrected for activity lost to urine. In the fasting state, EGP equals the glucose disappearance rate when plasma glucose is steady, and during a clamp/^18^F-FDG study, EGP can be calculated by subtracting the glucose infusion rate from the glucose disappearance rate during the steady state [80]:

(4) $$\mathrm{EGP}\;={\;R}_{d}+V_{\mathrm{glucose}}\times \frac{\Delta_{\mathrm{glucose}}}{\Delta_{T}}-\mathrm{GIR}\;$$

where R_d_ is the rate of disappearance, and GIR is the glucose infusion rate. GIR is corrected by a space correction [82] where V_glucose_ is the estimated glucose distribution volume (0.19 L/kg), Δ_glucose_ is the change in glucose from ^18^F-FDG injection to the end of sampling (mmol/L) and Δ_T_ is the time of ^18^F-FDG injection to the end of sampling (min).

Glucose disappearance rate (R_d_) is calculated using ^18^F-FDG clearance corrected by tracer lost to urine [80]:

(5) $$R_{d}=\frac{{\mathrm{dose}}_{\mathrm{FDG}}-{\;\mathrm{urine}}_{\mathrm{FDG}}}{{\mathrm{AUC}}_{\mathrm{FDG}}}{*\mathrm{avg}}_{\mathrm{glucose}}\;$$

where dose_FDG_ is the activity of the injected ^18^F-FDG, urine_FDG_ is ^18^F-FDG secreted to urine from the tracer injection until voiding bladder at the end of the study, AUC_FDG_ is the area under the curve representing ^18^F-FDG from the tracer injection to infinity and avg_glucose_ is the average glycemia during the interval between the time of ^18^F-FDG injection and the end of sampling.

An important consideration for the EGP measurement from a bolus ^18^F-FDG study is that the measurement time for the tracer blood curve is sufficiently long to reliably estimate ^18^F-FDG clearance from AUC_FDG_.

### 2.4. Measuring Hepatic GU: Effects of Obesity and Weight Loss

Obesity [43,44] and type 2 diabetes (T2DM) [83] have a negative effect on insulin-stimulated liver GU, whereas weight loss by bariatric surgery (BS) increases liver GU [44]. This impairment in insulin stimulation of liver GU measured by ^18^F-FDG-PET, which indicates decreased hepatic glucokinase activity, is in line with research showing lower UDP-flux during hyperinsulinaemia with intravenous or enteral glucose delivery [84,85] and postprandial glycogen synthesis rate [86] in patients with T2DM. In fact, in a study in rats, glucokinase activity/translocation has been shown to be the rate-controlling step in insulin-stimulated glycogen synthesis [87].

### 2.5. Measurement of Liver Perfusion

**Figure 3:**
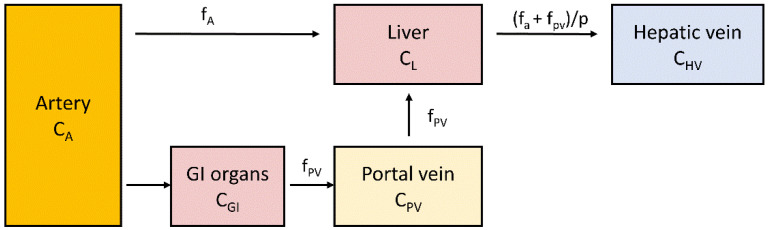
Schematic representation of a single compartment model of liver perfusion. C_A_, C_GI_, C_PV_, C_L_ and C_HV_ represent ^15^O-H_2_O concentrations in arterial, gastrointestinal, portal vein, liver and hepatic vein compartments: f_A_ represents perfusion from the artery, and f_PV_ represents portal vein perfusion; *p* is the partition coefficient of water in the tissue. Tracer concentration in portal vein can be measured either directly or derived from arterial input by taking into account delay and dispersion caused by blood passing through gastrointestinal organs [90,91].

Liver blood flow can be measured using positron emitting oxygen-15 labelled water (^15^O-H_2_O), a method that provides reliable information about tissue perfusion. This is possible thanks to the characteristics of this tracer being diffusible and not becoming metabolized or trapped. The labelled water diffuses rapidly from capillaries to extra- and intracellular spaces and back to circulation. ^15^O has a short half-life of only 122 s, which allows multiple measurements in a short period of time. This is a major improvement over Xenon-133 (half-life 5.3 days), a tracer earlier employed for blood flow measurements [88]. A study of liver blood flow needs to take into account the dual input of blood flow where the peak of the time–activity curve in the portal vein is delayed and dispersed compared to the hepatic artery [60,89,90]. Liver perfusion can be quantified by using a one-tissue compartment model where the tissue compartment receives input from hepatic artery and portal vein (Figure 3). If the arterial input function is obtained by sampling from a peripheral artery, the time-activity curve needs to be corrected for time delay between the sampling site and liver tissue.

In addition to ^15^O-H_2_O, various other tracers can be used to assess liver perfusion because of their highly effective clearance from blood to hepatocytes during the first minutes of tracer injection. This makes it possible to estimate hepatic perfusion with metabolic tracers such as ^18^F-FDG or 3-O-^11^C-methylglucose, although hepatic perfusion estimated using K_1_ from a compartment model had some tendency to overestimate perfusion in a pig study [59].

In a BS study [92], we showed that liver blood arterial blood flow (per unit volume) at the fasting state increased in patients with morbid obesity compared to non-obese controls, whereas there was no difference between the groups in portal blood flow. Blood flow was further increased after BS per unit volume but not changed per whole organ as liver volume decreased.

In another study [93], we found that there were no differences in basal hepatic blood flow between lean controls and patients with morbid obesity, but the hepatic blood volume was higher in morbid obesity due to organ size. In both groups, a mixed meal did not change blood flow but reduced hepatic blood volume about 10%. In this study, BS reduced blood flow in hepatic artery at fasting with no change in portal blood flow or hepatic blood volume. Portal blood flow after a mixed meal was increased after BS. BS induced a reduction in hepatic blood volume during gastrointestinal peptide infusion whereas GLP-1 infusion decreased portal vein blood flow and increased hepatic arterial flow in lean controls. [93]

We have previously shown that hepatic blood flow measurement has potential in the diagnosis of acute mesenteric ischemia [94]. Acute mesenteric ischemia has high mortality rates despite the availability of an effective treatment because the condition is difficult to diagnose at early stages based on clinical and laboratory findings or other imaging methods [94].

### 2.6. Measurement of Hepatic Fatty Acid Uptake

Hepatic fatty acid uptake (HFAU) can be measured with PET by using ^11^C-palmitate or the ^18^F-labelled fatty acid analogue 14(R,S)-[^18^F]fluoro-6-thia-Heptadecanoic acid (^18^F-FTHA).

**Figure 4:**
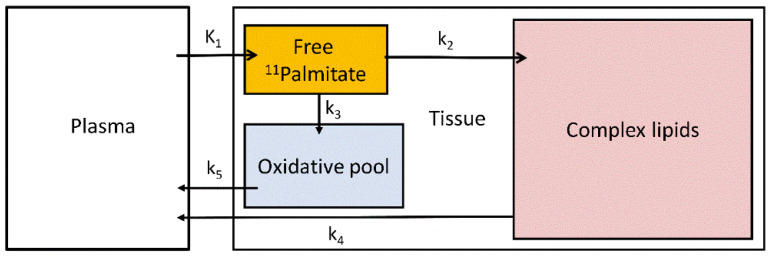
Three-tissue compartmental model of ^11^C-palmitate kinetics. K_1_ and k_2–5_ are the transfer rate constants between plasma, tissue free ^11^C-palmitate pool, oxidative pool and complex lipids.

Palmitate is a physiological substrate of the liver and one of the most common fatty acids stored in the adipose tissue and in circulation in humans [95]. Hepatic ^11^C-palmitate kinetics can be modelled with a three-tissue compartment model representing free ^11^C-palmitate, ^11^C-palmitate bound in lipids and ^11^C-oxidative metabolites [96] (Figure 4).

^18^F-FTHA differs from real fatty acids due to the sulfur heteroatom substituting the sixth carbon in the fatty acid carbon chain [97]. The presence of sulfur atom causes the metabolism of ^18^F-FTHA to stop after two cycles of beta oxidation, and ^18^F-labels from the metabolites of this process are trapped in the tissue [97]. ^18^F-FTHA is bound to phospholipids and glycerol esters [97,98], albeit fractional esterification into triglycerides has been shown to be impaired compared to ^14^C-palmitate [99] and in response to insulin [98,99]. The property of efficient trapping in the tissue allows the use of the Patlak graphical model to measure tissue uptake between 10 and 32 min from tracer injection before tracer loss from the tissue starts to bend the curves at later time points [100].

We have studied the effect of very-low calorie diet on hepatic fatty acid uptake, reporting a decrease of ~26% in HFAU following weight loss [101]. In another study, we studied hepatic fatty acid uptake in conjunction with blood flow and liver fat content (with MRS) in morbidly obese and lean individuals and the effect of BS [44]. Patients with obesity had significantly higher HFAU compared to the lean controls. Contrary to the very-low calorie diet study, in this study, 6-months following BS, HFAU remained high, because of the strong catabolic state induced by metabolic surgery. On the contrary, following BS, the liver’s fat content was markedly decreased. Taken together, it was suggested that in post-surgery settings, the large amounts of fatty acids uptaken by the liver, are not stored in the liver but are used for oxidation to provide energy.

The effect of the sodium-glucose cotransporter-2 inhibitor (SGLT2-i), dapagliflozin, on HFAU was also recently evaluated by us [102]. In that study, we found that hepatic fatty acid uptake was increased by dapagliflozin versus placebo.

### 2.7. Liver ^11^C-Acetate: Applications 

Imaging with ^11^C-labelled acetate (1-^11^C-acetate) can provide information about blood flow, oxidation, and fatty acid synthesis in the liver. The initial build-up of the tracer is related to tissue blood flow and washout describes CO_2_ production; in addition, part of the tracer is incorporated to amino acids [103]. About 30 min after 1-^11^C-acetate injection most tracer in the tissue is bound to long-chain fatty acids. This irreversible uptake representing de novo fatty acid synthesis can be measured by using the Patlak-plot similarly to GU and HFAU [103].

### 2.8. ^18^F-FDG-PET and Nonalcoholic Fatty Liver Disease

Nonalcoholic fatty liver disease (NAFLD), recently renamed as metabolic associated fatty liver disease (MAFLD), is the leading cause of chronic liver disease worldwide [104]. MAFLD is associated with cardiovascular, metabolic, digestive, and neurodegenerative diseases, but when there is concomitant presence of liver inflammation (i.e., steatohepatitis (NASH)), the progression to these diseases is markedly accelerated [105]. Since inflammatory cells are characterized by high glucose-avidity, it would be expected that in the presence of NASH, ^18^F-FDG-PET would show high liver GU rates [106]. However, the available literature has yielded contrasting results.

Keramida et al. reported that hepatic steatosis is associated with increased hepatic ^18^F-FDG uptake [107]. On the contrary two previous studies have reported decreased liver GU rates (evaluated using SUV) in patients with liver steatosis [108,109]. A likely explanation to these apparently contrasting results is that increased fat content reduces uptake per liver volume unit as fat does not contribute to GU but uptake per fat-free liver tissue increases [72]. Thus, measuring liver fat content and volume with computed tomography (CT) or MR [110] should be considered when studying liver metabolism with PET as it can be easily performed with combined PET-CT/MR tomographs.

Two recent reports that used compartmental modelling of ^18^F-FDG in the liver and also evaluated liver histology reported an inverse correlation between hepatic inflammation grades and liver blood flow [111,112]. However, in these studies, only relative indices but not the absolute rate of hepatic glucose uptake (HGU) were assessed.

Recently, Guzzardi et al. studied Zucker rats (thus, a rat model of leptin-receptor deficiency). They reported that liver glucose uptake was proportional to the degree of fat accumulation and tissue inflammation and was able to dissect healthy from NAFLD and NAFLD/NASH livers. However, the most severe NASH livers showed a decline in glucose uptake [113]. Taken together, whether histology-proven NASH in humans is associated with enhanced HGU remains to be proven.

### 2.9. ^11^C-Acetate-PET and NAFLD

In a retrospective study of prostatic carcinoma patients, hepatic ^11^C-acetate uptake was positively associated with CT-measured hepatic fat content [114]. The study showed the potential for ^11^C-acetate-PET as a diagnostic tool for NAFLD, although the lack of biopsy-based measurement of fat content, a healthy control group and female participants suggest that more thorough investigation is needed to assess whether ^11^C-acetate-PET would be useful for NAFLD diagnostics.

## 3. PET Metabolic Tracers in Hepatic Malignancies

### 3.1. ^18^F-FDG

^18^F-FDG PET is an established imaging method used in the oncology setting for staging, restaging and follow-up of a wide spectrum of malignant diseases. Cancer cells usually present with a disorder of one or more glycolytic pathways, including the higher expression of glucose transporters and hexokinase than normal cells [115,116]. The increased glucose metabolism in cancer tissues results in increased ^18^F-FDG uptake assessed by PET imaging. The degree of uptake is associated with histological characteristics, tumour differentiation and intratumoural alterations [117]. 

### 3.2. ^18^F-FDG in Hepatocellular Carcinoma

The most common primary malignancy that affects the liver is hepatocellular carcinoma (HCC). Accurate staging of HCC is crucial as only patients with small tumours without distant metastases would benefit from liver resection or transplantation as a curative treatment. HCC cells present with varying expression of ^18^F-FDG uptake due to the lower expression of glucose transporters and hexokinase in well-differentiated tumours compared to other hepatic malignancies [118]. For this reason, the sensitivity of ^18^F-FDG PET in detecting primary HCC is relatively limited, with reported values of 50–70% [119,120,121]. However, it has been demonstrated that poorly differentiated HCCs show significantly higher glycometabolic activity [119]. In view of this, ^18^F-FDG PET imaging might have an important prognostic value, reflecting the potential of tumour progression and recurrence [122]. Moreover, ^18^F-FDG PET has a promising role in staging HCC because of the high accuracy in detecting extrahepatic metastases, which would possibly affect patient management and treatment selection [122,123]. 

### 3.3. ^18^F-FDG in Intrahepatic Cholangiocarcinoma

Intrahepatic cholangiocarcinoma (ICC), a relatively rare neoplasm that arises from the bile duct epithelium, is the second most common primary liver malignancy after HCC. ICC cells have a higher expression of glucose transporter and hexokinase than HCC, resulting in typically high ^18^F-FDG uptake that is particularly prominent in nodular or mass-forming cholangiocarcinoma [120,124]. ICC tends also to show ring-shaped uptake, corresponding to peripheral rim enhancement visible on CT and/or MRI [124]. ^18^F-FDG PET has demonstrated to be an accurate diagnostic tool for the detection of occult metastasis or characterization of indeterminate lesions, having a major influence in clinical decision making in patients with ICC [125]. Moreover, ^18^F-FDG PET imaging has shown high sensitivity in the detection of primary tumour and nodal metastases [125,126].

### 3.4. ^18^F-FDG in Liver Metastases

The liver is involved more often with metastatic disease than primary tumours. The main indication of ^18^F-FDG PET in liver imaging is, in fact, the evaluation of metastasis arising from tumours of the gastrointestinal tract, lung, breast, pancreas and sarcoma [127,128]. Liver metastases are potentially curable with hepatic resection, and their accurate detection and characterization is essential to determine the proper treatment strategy.^18^F-FDG PET-CT is highly sensitive and specific in diagnosing liver metastases, which generally show high tracer uptake [128]. Moreover, ^18^F-FDG PET has a prognostic role in follow-up on patients who underwent surgical or ablative procedures, since it is sensitive in detecting residual or recurrent diseases. The liver is the most frequent site of metastases in colorectal cancer (85). There is solid evidence on the performance of ^18^F-FDG PET in patients with colorectal cancer. Studies have shown greater accuracy than CT or MRI in primary staging and recurrence settings, as well as in the evaluation after local and ablative therapies [129,130,131].

**Figure 5:**
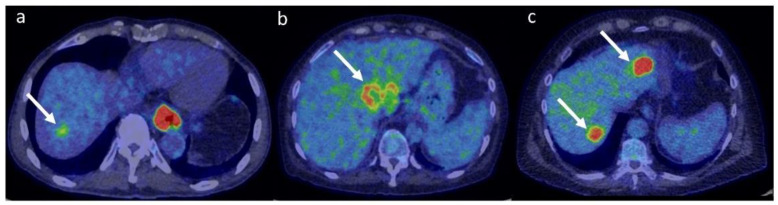
Fused ^18^F-FDG PET/CT representative images of HCC (**a**), ICC (**b**) and liver metastases from colorectal cancer (**c**). Note the relatively mild uptake in HCC (SUVmax 4.2 g/mL), the ring-shaped uptake in ICC (SUVmax 7.8 g/mL) and the high uptake in metastatic lesions (SUVmax 15.3 g/mL). PET colour scale 0–7. The patient represented in panel (**a**) had a concomitant oesophageal cancer.

It is worth mentioning that some liver metastases may present with low FDG-uptake. These might include well-differentiated malignancies, such as pancreatic or lung adenocarcinomas [132,133]. Moreover, tumours with cystic or mucinous components might present with reduced metabolic activity due to low cellularity [134]. Figure 5 shows representative ^18^F-FDG PET images of hepatocellular carcinoma, cholangiocarcinoma and liver metastases.

### 3.5. ^11^C-Acetate in Liver Malignances

Despite ^18^F-FDG being the most successful PET metabolic tracer used in oncology, ^11^C-acetate has also shown some promising applications in imaging liver malignancies. The incorporation of ^11^C-acetate in cancer cells is mostly connected to the upregulation of free fatty acid synthesis [135]. Considering the varying sensitivity of ^18^F-FDG PET in detecting HCC, ^11^C-acetate might be used as a complementary tracer. Tumour uptake of HCC with both tracers might be correlated to histologic differentiation, as well-differentiated tumours seem to be ^11^C-acetate-avid, while in poorly differentiated HCC or in more advanced stages, the metabolic substrates in tumour cells shift to glycolysis and, thus, ^18^F-FDG uptake [136,137]. Moreover, ^11^C-acetate seems to be specific for the detection of HCC, as cholangiocarcinoma or secondary metastases might not show tracer uptake [136]. Therefore, a dual-tracer approach may be useful in the appropriate staging of HCC tumours and in the differential diagnosis of unknown liver lesions [136,137,138]. These preliminary data are, however, limited, and further studies are needed to assess the potential role of ^11^C-acetate in this setting.

### 3.6. Limitation of PET Technology in Imaging Liver Malignancies

The main technology-related limitation of PET imaging in the assessment of liver malignancies is the relatively low sensitivity in detecting small lesions. This is usually related to the spatial resolution of the camera, which is typically 5 mm in current scanners. The limited spatial resolution can lead to a problem with the phenomenon known as partial volume effect, when radioactivity in the volume of interest (VOI) of small lesions (<1 cm in diameter) is detected as being spread to the surrounding tissues, causing an underestimation of activity concentrations in the volume of interest [139]. In addition, VOI may contain also other tissues when the edges of a metabolically active lesion do not coincide with VOI’s voxel contours [139]. The sensitivity of ^18^F-FDG PET/CT for the detection of HCC according to tumour size has been reported to be 27% for lesions measuring 1–2 cm, whereas the values were 48% and 93% for lesions of 2–5 cm and >5 cm, respectively [140]. Studies have also demonstrated that ^18^F-FDG PET/CT is accurate in the detection of hepatic metastases >1 cm in diameter; however, sensitivity can decrease significantly in lesions <1 cm [141,142]. Respiratory motion can also limit the detectability of small liver lesions, especially when located in the cranial area of the liver close to the diaphragm. Moreover, technology-specific limitations such as the misregistration of PET and CT datasets, attenuation correction and truncation artefacts could affect the sensitivity of liver-related PET imaging [143].

## 4. Discussion

PET imaging provides the unique opportunity to study metabolic rates at the tissue level non-invasively. The previous gold standard AV differences technique has been lately abandoned due to its invasive nature and the fact that it could not exclusively assess liver metabolic rates, but the substrate uptake rates from the splanchnic area were reported. A major discrepancy between the AV differences technique and ^18^F-FDG PET results has been that whereas DeFronzo et al. [144] did not find an effect of insulin on splanchnic GU at 5 mmol/L euglycemia, Immonen et al. [44] reported an increase in hepatic GU from the fasting to the insulin clamp state. Moreover, the GU rates reported in AV differences studies from the splanchnic area would be much higher to those reported with ^18^F-FDG PET for liver GU. This difference is likely at least partly explained by GU into other splanchnic organs than the liver, which has been shown to be considerable in dogs [42] and humans [145,146,147]. Quantitative PET studies of the liver are complicated by its dual input function. Blood supply from the hepatic artery has the same tracer concentration as all other arteries, with a very sharp curve peak in the case of bolus administration of the tracer. However, most of the blood supply to the liver comes via a portal vein; the tracer is first distributed to the intestines, spleen, pancreas, and gallbladder, and as a result, the concentration peak is dispersed, delayed, and possibly affected by the metabolic processes in splanchnic organs: Depending on the tracer, AUC, or the fraction of label-carrying metabolites, it may be different in arterial blood and portal vein. A study employing continuous ^18^F-FDG infusion suggested that the continuous delivery may provide more robust estimates of hepatic glucose metabolism than bolus injection [64]. Alternatively, portal input may be measured directly from PET images for studies of liver metabolism [148] if metabolites of the tracer are not released into circulation as is the case with ^18^F-FDG or if the amount of metabolites is carefully determined [149]. The relatively small diameter of the vein and movement due to breathing render deriving image-based portal vein input function a laborious task to perform manually. Computational methods for (semi)automatic segmentation [150] and the development of motion correction methods [151,152,153] will likely make obtaining image-derived portal vein input function more feasible in the future. It can be speculated that ^18^F-FDG6P trapping in the liver would reflect the distribution of labour across the liver acinus where periportal hepatocytes are more focused on gluconeogenesis and glucose release, and pericentral hepatocytes focus on glucose uptake and glycolysis [16]. Thus, novel microvascular compartment models taking into account tracer gradients across the sinusoids and tracer backflow and reuptake [154] have potential for detailing the differences in hepatic cellular metabolism in vivo across the liver acinus in the future.

^18^F-FDG PET/CT is an accurate and sensitive method to assess primary and metastatic liver malignancies, despite some challenges given by the physiological tracer activity in liver tissue and the varying uptake of liver tumours depending on histology [123,130,155]. In the near future, the development of radiomics, a textural analysis of PET images, might provide new information to better characterize liver tumours, predict prognosis and potentially select management strategies [156]. Moreover, the development of more advanced and sensitive PET technology, together with the increasing use of PET/MRI scanners, might improve the early detection of small liver lesions and lead to a better management of oncological patients.

More general limitations to the use of PET for measuring tissue specific metabolism and blood flow include the high cost of tomographs and tracer production as well as the need for highly trained personnel. The radiation dose emitted by the tracers limits the repeated use of PET especially with ^18^F-FDG. New PET tomographs with a long axial field-of-view (FOV; 70–200 cm) provide intriguing opportunities for measuring the simultaneous metabolism of different organs, enabling a more effective evaluation of inter-tissue dynamics of metabolism. An important limitation of the currently used 15–30 cm FOV PET tomographs has been their poor ability to detect coinciding emitted photons (<1%) due to their narrow FOV. Thus, an important strength of the long axial field-of-view tomographs is their improved sensitivity, which is estimated to be 4–5 times higher with a 200 cm FOV tomograph when imaging a single organ and up to 40 times higher for the entire body compared to conventional tomographs that are currently in use [157]. Depending on the study question, the possibility to record radioactivity signals from various organs simultaneously and the increased sensitivity of the long axial FOV scanners may allow researchers and clinicians to reduce the scanning time or radiopharmaceutical dose considerably [158].
